# The Role of MicroRNAs in Endothelial Cell Senescence

**DOI:** 10.3390/cells11071185

**Published:** 2022-03-31

**Authors:** Jovana Nikolajevic, Nazila Ariaee, Aaron Liew, Shadi Abbasnia, Bahare Fazeli, Miso Sabovic

**Affiliations:** 1 Department of Vascular Diseases, University Medical Center, 1000 Ljubljana, Slovenia; miso.sabovic@kclj.si; 2 Allergy Research Center, Mashhad University of Medical Sciences, Mashhad 1696700, Iran; ariaeen931@mums.ac.ir; 3 Department of Medicine, National University of Galway, H91 CF50 Galway, Ireland; aaron.liew@nuigalway.ie; 4 Immunology Research Center, Inflammation and Inflammatory Diseases Division, Mashhad University of Medical Sciences, Mashhad 1696700, Iran; abbasniash9@mums.ac.ir; 5 Vascular Inflammation Research Center, Mashhad University of Medical Sciences, Mashhad 1696700, Iran; bahar.fazeli@gmail.com; 6 Medical Faculty, University of Ljubljana, 1000 Ljubljana, Slovenia

**Keywords:** senescence, endothelial cells, ageing, micro-RNA, endothelial dysfunction, age-related diseases

## Abstract

Cellular senescence is a complex, dynamic process consisting of the irreversible arrest of growth and gradual deterioration of cellular function. Endothelial senescence affects the cell’s ability to repair itself, which is essential for maintaining vascular integrity and leads to the development of endothelial dysfunction, which has an important role in the pathogenesis of cardiovascular diseases. Senescent endothelial cells develop a particular, senescence-associated secretory phenotype (SASP) that detrimentally affects both surrounding and distant endothelial cells, thereby facilitating the ageing process and development of age-related disorders. Recent studies highlight the role of endothelial senescence and its dysfunction in the pathophysiology of several age-related diseases. MicroRNAs are small noncoding RNAs that have an important role in the regulation of gene expression at the posttranscriptional level. Recently, it has been discovered that miRNAs could importantly contribute to endothelial cell senescence. Overall, the research focus has been shifting to new potential mechanisms and targets to understand and prevent the structural and functional changes in ageing senescent endothelial cells in order to prevent the development and limit the progression of the wide spectrum of age-related diseases. The aim of this review is to provide some insight into the most important pathways involved in the modulation of endothelial senescence and to reveal the specific roles of several miRNAs involved in this complex process. Better understanding of miRNA’s role in endothelial senescence could lead to new approaches for prevention and possibly also for the treatment of endothelial cells ageing and associated age-related diseases.

## 1. Introduction

Cellular senescence is a complex, dynamic process comprised of replicative senescence (the state of the irreversible arrest of growth and loss of replicative capability) alongside structural and functional changes and gradual deterioration of cellular function [1].

Senescent cells undergo quite substantial changes in their structure and function: DNA damage, chromatin remodelling, mitochondrial dysfunction and changes in protein expression and secretion resulting in the development of the senescence-associated secretory phenotype (SASP) [2]. The main characteristic of this phenotype is a dysregulated production of proinflammatory cytokines, chemokines and growth factors (IL-1a, IL-1b, IL-6, IL-8, IL-18, CCL-2, TNF-a) as well as proteases (MMP-1, -2, -3, -7, -8, -9, -10, -12, -13, and -14 [3,4]. SASP contributes to the propagation and amplification of senescence to other cells and organs, both surrounding and distant, thereby facilitating the ageing process and development of age-related disorders. The accumulation of senescent cells, which were not successfully cleared by the ageing immune system, leads to progressive morphological and functional deterioration of tissues and organs, ultimately resulting in the development of age-related diseases.

During senescence, endothelial cells (ECs) undergo the same phenotypic transition leading to the development of SASP. Changes in structure and function, resulting from that process, represent a very important contribution to the development of endothelial dysfunction and age-related cardiovascular (CV) diseases such as atherosclerosis [5]. Senescent ECs represent an important source of both local and systemic, chronic low-grade inflammation [6]. Another hallmark of the senescent phenotype is mitochondrial dysfunction, leading to increased production of reactive oxygen species (ROS) [7]. Both inflammation and oxidative stress have been recognized for their contribution to the development of endothelial dysfunction [7]. Increased ROS production causes oxidative damage in DNA as well as other molecules (proteins and lipids) leading to further progression of cellular senescence [8]. Pathological neo-angiogenesis, as shown in the retinopathy model, is the overall effect of the development of SASP [9].

By inducing permanent growth arrest, ageing does not just decrease endothelial reparative ability, considered essential for vascular integrity, wound healing, inflammation, tumourigenesis and collateral blood flow development, but is also associated with derangement of endothelial function [10]. Senescence diminishes not only endothelial barrier function but also its metabolic, haemodynamic, anti-inflammatory and anti-thrombotic function [11]. Endothelial dysfunction is well recognised as an important contributor to the complex pathophysiology of many CV diseases [12]. Furthermore, an increasing body of evidence has highlighted the importance of endothelial dysfunction in the pathophysiology of several ageing-related diseases [13,14,15,16]. Currently, research focus has been shifting to new potential targets and mechanisms of prevention of the structural and functional changes in ageing endothelial cells in order to prevent the development and limit the progression of the wide spectrum of age-related diseases.

## 2. Factors Inducing Cellular Senescence

Replicative senescence has been recognised as the limited ability of cells to replicate (Hayflick limit) and is associated with progressive telomere shortening. The senescent phenotype could also develop in response to cellular stress and damage, known as “stress-induced premature senescence” [17]. This form is not associated with progressive telomere shortening but with non-telomeric DNA damage and persistent mitogenic stimulation [18].

To date, numerous factors have been recognised for their ability to induce the development of the senescent phenotype: mitogens, inflammatory molecules, oxidants and antioxidants, angiotensin II, nitric oxide (NO), high glucose levels and advanced glycation end-products (AGEs) or exposure to ionising radiation [19,20,21,22]. Most of these factors act either by modulating oxidative stress levels, telomerase activity and telomere integrity or by causing oncogene activation or genomic DNA damage [22,23]. Moreover, some factors may induce senescence by acting at the epigenetic level, by dysregulating DNA methylation or histone modification [24]. Taken as a whole, among all listed factors, the strongest evidence supports the utmost importance of reactive oxygen species (ROS) in the induction of cellular senescence. Oxidative stress can induce senescence by acting directly on telomeres (also in a length-independent manner) or by acting on genomic or mitochondrial DNA [19]. Extensive research on potential mechanisms by which ROS can induce senescence has revealed a variety of complex functions and involvement in many different signalling pathways: reduction of sarco/endoplasmic reticulum Ca^2+^ -ATPase activity, induction of matrix metalloproteases (MMPs) expression, regulation of mammalian target of rapamycin (mTOR) complexes, inhibition of sirtuins activity, regulation of signalling pathways (e.g., p16INK4a/pRB and p53/p21), or modulation of cytokine production [25].

Recent studies have elucidated a novel mechanism of senescence induction, resulting from a complex interplay of ROS and microRNA (miRNA) expression and modulation of gene silencing. This interaction appears to be bidirectional—ROS have been reported to modulate the production or expression of different miRNAs. In turn, some signalling pathways that regulate intracellular oxidative metabolism and ROS production, are controlled by specific miRNAs [26].

## 3. miRNAs and Endothelial Senescence

MiRNAs are small, single-stranded, non-coding RNAs that modulate gene expression at the post transcriptional level. MiRNAs act mostly by directly binding to the 3′ untranslated region (3′ UTR) of target messenger RNA (mRNA) sequences, thus inhibiting translation and/or promoting the degradation of target mRNA. In rare cases, miRNAs could also promote mRNA translation [27,28]. In general, miRNAs are not gene-specific—all miRNAs have more than one target gene, and most target genes are regulated by more than one miRNA [29]. To date, miRNAs have been found in small membranous vesicles called exosomes, in apoptotic bodies, high- and low-density lipoproteins (HDL/LDL), RNA-binding proteins, as well as circulating in human serum [30,31,32,33,34]. The existence of miRNAs has also been confirmed in other human body fluids, such as serum, plasma, saliva, breast milk, urine and cerebrospinal fluid [35]. These findings raised the question of whether miRNAs have other functions besides gene expression modulation, e.g., mediation of cell–cell communication.

As a result of the increasing knowledge about miRNAs and their functions, research interest in the development of new diagnostic procedures based on the detection and quantification of specific miRNAs is ever greater. Studies have reported that the presence and quantity of specific miRNAs could serve as a potential biomarker of disease progression [36]. MiRNAs have also been suggested as good candidates for the characterisation of genomic alterations in solid cancer, analogous to parallel sequencing of circulating tumour DNA released from cancer cells into the plasma, the concept called “liquid biopsy” [35].

**Figure 1:**
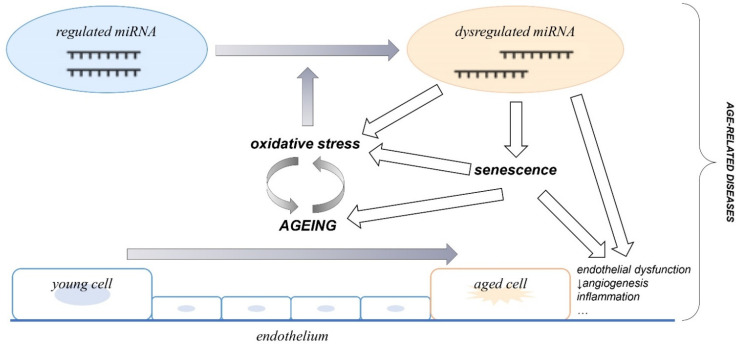
The role of dysregulated miRNAs in the pathophysiology of endothelial cell senescence and ageing, and their contribution to the development of age-related diseases.

Most studies report a decreased abundance of miRNAs in ageing, while evidence that the stability of miRNAs changes with age is still lacking [26]. Moreover, the expression of numerous miRNAs changes with age, suggesting complex ageing-associated alteration of miRNA biogenesis, which could have an important role in ageing-related pathophysiologic changes [37]. A schematic diagram describing the suggested essential role of dysregulated miRNAs in the process of EC senescence and ageing, and the associated development of age-related diseases is presented in Figure 1.

The aim of this review is to provide an insight into miRNAs that could have an important role in the modulation of processes involved in senescence pathophysiology and the mechanism by which that modulation is achieved. The miRNAs included in this review were selected on the basis of the existing knowledge about processes involved in the pathophysiology of cellular senescence and the development of endothelial dysfunction and documented or suspected role that particular miRNA could play in their modulation. The potential mechanism by which particular miRNA could take part in senescence pathophysiology, its target genes/signalling pathways and the types of cells used in the research are listed in Table 1.

## 4. MiRNAs and Their Specific Roles in Endothelial Cell Senescence

### 4.1. miR-126

Studies of old Human Umbilical Vein Endothelial Cells (HUVEC) have shown that the most abundant miRNAs in aged EC are miR-100, miR-21, and miR-126. MiR-126 is the most expressed miRNA in EC, particularly in young cells, and the only miRNA in vertebrates whose expression is restricted to EC [41]. Increasing evidence supports its role in angiogenesis and inflammation processes. Overexpression of miR-126 can block activation of the PI3K/Akt signalling pathway, thus inhibiting ischemic angiogenesis and tumour growth [38]. MiR126 overexpression has been reported to have a protective effect in ischemia/reperfusion injury, probably due to its antioxidative and anti-inflammatory effects in HUVECs due to the activation of the SIRT1/Nrf2 signalling pathway resulting in a decreased level of oxidative stress and inflammation [39,40]. It has been reported that miR-126 reduces the expression of vascular cell adhesion molecule 1 (VCAM-1) in ECs followed by reduced leukocyte–EC interaction in response to TNF-α [41,42]. On the other hand, decreased expression of miR-126 is associated with increased mRNA and protein levels of VEGF-A, which are known to modulate migration and formation of vessel lumen as well as the expression of IL-10 [38,42].

When two mature miRNAs are generated from the opposite arms (5′ or 3′) of the same miRNA precursor, the mature miRNAs are denoted with a -5p or -3p suffix, respectively. The level of miR-126-3p and miR-126-5p in plasma was shown to be decreased in patients with diabetes mellitus (DM) as well as in patients with severe coronary artery disease [78,79]. Circulating levels of miR-126-3p increase with age in healthy people but not in people with DM, leading to the hypothesis that the absence of that senescence-associated compensatory mechanism could, at least partially, be responsible for higher CV risk in patients with DM [80].

Besides miR-126, the expression of numerous miRNAs is regulated by Nrf2 (nuclear factor erythroid 2-related factor 2): miR-21, miR-100, miR-34, miR-146, miR-217, let-7b and many others [29]. Nrf2 has been known to represent a hallmark of lipid load and the inflammatory microenvironment of atherosclerotic plaques [29]. Besides modulating the expression of miRNAs and antioxidant genes, Nrf2 regulates the expression of genes involved in the metabolic control, repair and degradation of damaged macromolecules of the cell [81]. Nrf2 also acts as a mediator of oxidised phospholipid signalling as well as the cellular response to hypoxic, oxidative, or electrophilic stress [43,82].

To summarize: age-related increase in plasma miR126 could be related to senescence pathophysiology by modulating hypoxic, inflammatory and antioxidative response of EC as well as leukocyte adhesion and repair/degradation of damaged molecules in EC.

### 4.2. miR-21 and miR-100

Besides miR-126, both miR-21 and miR-100 have high level of expression in EC and are regulated by Nrf2. The expression of both miR-21 and miR-100 has been reported to be increased in senescent cells. After exposure of EC to proatherogenic stimuli, the expression of both miRNAs decreases, probably due to decreased Nrf2 expression [29,83].

It has also been suggested that the presence and quantity of miR-21 could serve as a potential biomarker to assess the endothelial senescence stage [44]. Furthermore, in patients with coronary artery disease (CAD) miRNA levels in the pericardial fluid could have even better diagnostic values than total fluid miRNA values [84]. Notably, the levels of both miR-21 and miR-100 were lower in the CAD group compared to the control group. After patients had been further divided into subgroups on the basis of the severity of heart failure (HF) symptoms (NYHA I-IV), levels of miR-21 increased while levels of miR-100 decreased in accordance with the severity of HF symptoms [84].

Both miRNAs activate the VEGFA/MYC pathway thus playing an important role in angiogenesis, proliferation and increased metabolic activity of ECs [29]. However, they act in an opposite way as activation of the pathway requires miR-21 upregulation and miR-100 downregulation [29]. Inhibition of both miR-21 and miR-203a results in inhibition of the AMPK-p53/p16 signalling pathway thus preventing mitochondrial dysfunction [44].

Recent studies revealed the ability of the endogenous protein kallistatin, a tissue kallikrein-binding protein and a serine proteinase inhibitor, to reduce oxidative stress and inflammation and enhance the mobility and function of endothelial progenitor cells (EPCs) in animal models [45]. Subsequent studies confirmed that kallistatin prevents endothelial–mesenchymal transition by blocking TGF-β1-mediated miR-21 synthesis and reduces the production of ROS by stimulating the expression of antioxidant genes eNOS, SIRT1 and FoxO1 [46]. According to existing evidence, miR-100 has an atheroprotective role as it inhibits the proliferation of EC and migration of vascular smooth muscle cells [47]. Research on a mouse model of atherosclerosis supports this finding, as miR-100-5p overexpression was shown to attenuate atherogenesis and decrease plaque area by 45% [48].

Thus, miR-21 and miR-100, whose expression increases with age, could modulate EC senescence by modulation of angiogenesis and proliferation of EC, migration of VSMC as well as mitochondrial functioning and ROS production.

### 4.3. miR-34a

MiR-34a is overexpressed in senescent cells (endothelial, cardiac, splenic) [85]. Extensive research on the role of miR-34a in the regulation of cell senescence confirmed its involvement in different signalling pathways in senescence pathophysiology. Originally, miR-34a was considered a tumour suppressor miRNA and ectopic expression of miR-34a was reported to induce apoptosis in many cancers [86]. It attracted great attention after the discovery of its ability to upregulate the tumour suppressor p53 by downregulating the sirtuin 1 (SIRT1) gene [87]. A recent study identified both miR-34a and miR-29a/b/c as p53 downstream senescence mediators, as they inhibit cyclin A2 (CCNA2), independently of p53/p21 signalling [88].

SIRT1 is a member of the histone III deacetylase family, responsible for the deacetylation of lysine in histones and the conservation of DNA in the state of transcriptionally inactive heterochromatin [89]. Additionally, sirtuin-1 causes deacetylation of many regulatory proteins and transcription factors, including NF-κB and peroxisome proliferator-activated receptor γ (PPARγ), forkhead box O1 (FoxO1) and enhances NO production by deacetylating eNOS [49,50,51]. The effect of eNOS activation, observed after statin therapy, was attributed to an increased SIRT1 expression and direct stimulation of the Akt signalling pathway [51]. By modulating the activity of many different proteins, enzymes and transcription factors, SIRT1 is involved in a wide spectrum of atheroprotective mechanisms: regulation of thrombosis and fibrinolysis, inhibition of proprotein convertase subtilisin/kexin type 9 (PCSK9) and LDL reduction, protection from ischemia/reperfusion injury in cardiomyocytes, regulation of glucose metabolism, inflammation control and preservation of mitochondrial integrity [52]. Overexpression of miRNA-34a followed by SIRT1 downregulation may result in alteration of numerous CV risk factors and increased risk of CV diseases. Besides SIRT1, miR-34a overexpression suppresses the expression of both eNOS, and catalase [90]. Kallistatin was initially reported to inhibit miR-34a synthesis, as well as the expression of SIRT1, eNOS, and catalase in endothelial progenitor cells. A recent study reported that kallistatin itself does not have antioxidant effects at all, as all the antioxidative effects were mediated by miRNA-34a inhibition, confirming its central role in this signalling pathway [90].

Thus, overexpression of miR-34, observed in senescent EC, could promote senescence by modulating some of the key signalling pathways involved in senescence pathophysiology as well as thrombosis, fibrinolysis, NO production, glucose metabolism and inflammation.

### 4.4. miR-130a

MiR-130a was reported to be markedly downregulated in different types of ageing cells [91]. Studies consistently reported its role in cellular proliferation and angiogenesis, protection from ischemia/reperfusion injury. Experimental miRNA-130a overexpression was reported to increase proliferation and migration of EC and promote neovascularisation and angiogenesis [53]. These effects are mediated by the modulation of the expression of proangiogenic genes HOXA5 and MEOX2 [91]. MEOX2 also inhibits eNOS and decreases bioavailability of NO, thus affecting VEGF-induced angiogenesis and ischemia-induced neovascularisation [54].

Furthermore, miR-130a enhances hypoxia-induced smooth muscle cell proliferation and might be involved in the pathophysiology of pulmonary hypertension [55]. It has also been reported that miRNA-130a supplementation could attenuate cardiac dysfunction and remodelling following myocardial infarction [92].

A recent study reported significant upregulation of miRNA-130a in hypertrophic cardiomyopathy. Experimental in vivo inhibition of miR-130a significantly reduced cardiac fibrosis by modulating the expression of several fibrotic genes (collagens, Fn and CTGF), adhesion molecules and inflammatory genes, but a key target in this process appeared to be PPARγ [56].

To summarize: miR-130a, whose expression is decreased in senescent cells, could participate in senescence pathophysiology by modulation of ECs proliferation and migration, NO bioavailability and expression of fibrotic and inflammatory genes and adhesion molecules.

### 4.5. miR-222-221 Cluster

Genes encoding miR-222 and miR-221 are located on the X chromosome and share the same gene cluster. Consequently, those two miRNAs share some sequences, binding sites and target genes. Both miRNAs are highly expressed in human endothelial progenitor cells and also in quiescent EC, suggesting their important role in regulating endothelial development and function [60]. Serum levels of miR-221 and miR-222 were reported to be markedly deregulated in several vascular and metabolic diseases (hypertension, coronary artery disease, atherosclerosis and obesity) [93,94]. Extensive research has provided evidence of anti-proliferative, anti-migratory and pro-apoptotic effects in EC while their effects in vascular smooth muscle cells were reported to be quite the opposite [57]. The expression of miR-222 was reported to be increased in exercise-induced cardiac growth and acute heart injury models and promote cardiomyocyte proliferation [58].

Overexpression of both miR-221 and miR-222 was found to potentially interfere with cell cycle regulation, consequently affecting cellular growth and division, which is especially important in tumor biology. The mechanism by which miR-221 and miR-222 can alter cell cycle regulation is complex and involve several genes (p27, p57, PTEN and TIMP3). Overexpression of both miR-221 and miR-222 was found to decrease the expression of p27, tumor suppressor protein, by inhibition of p27 mRNA translation [59]. Furthermore, by activating the mTOR axis, inhibition of p27 was found to inhibit autophagy (enzymatic breakdown of a cell’s cytoplasm or damaged organelles or proteins) [95]. As those structures are known for their potential to trigger the complex process of apoptosis, inhibition of autophagy could lead to increased apoptosis, one of the hallmarks of pathological cardiac remodelling [95].

The miR-222-221 cluster also regulates some antioxidative and inflammatory pathways, implicating its complex role in vascular biology. Overexpression of miR-222-221 in human aortic EC is associated with increased production of ROS, probably by regulating PPARγ coactivator-1α [96]. MiRNA-221 suppresses NO synthesis and activates nuclear factor-kappa B (NF-κB) signalling in HUVEC by targeting the adiponectin receptor (AdipoR1) [97].

MiR-221 and miR-222 are markedly downregulated after exposure to inflammatory stimuli, suggesting their putative role in the modulation of inflammatory immune response. This hypothesis is supported by the finding that miR-222-221 inhibition increases inflammation and viral load upon viral myocarditis [98].

Thus, upregulation of miR-222-221 cluster, observed in senescent EC, could promote senescence by modulating cellular growth and division, autophagy/apoptosis, proliferation and migration of EC as well as NO and ROS production.

### 4.6. miR-200 Family

The miR-200 family consists of five members (miR-200a, -200b, -200c, -141 and -429), which are clustered and expressed as two separate transcripts (miR-200b-200c-429 and miR-200a-141), probably having similar target genes [99]. Found to be aberrantly expressed in different types of cancer, and downregulated in aggressive types of human tumours, members of the miR-200 family have long been the focus of oncological research. To date, studies have confirmed its role in the regulation of some of the most important processes in cancer biology such as migration, tumour cell adhesion, epithelial-mesenchymal transition and angiogenesis [100,101]. Translating the evidence from tumour biology to a wider spectrum of diseases with similar pathophysiologic features, researchers suspected its involvement in the pathophysiology of some CV diseases. A study comparing the expression of miR-200c in patients with bicuspid and tricuspid aortic valves without aortic dilation reported lower expression of miR-200c in patients with a bicuspid aortic valve, in parallel with higher expression of target genes ZEB1 and ZEB2, known to regulate endothelial-mesenchymal transition [61].

Despite extensive oncological research, little is known about the potential effects of members of this family on the CV system. The strongest body of evidence supports its involvement in the pathophysiology of insulin resistance and diabetes and its contribution to the development and progression of diabetic vascular complications [62]. Members of the miR-200 family downregulate SIRT1 leading to subsequent activation of p53 and inhibition of antioxidant enzymes, culminating in increased ROS production and induction of apoptosis [102]. The increased expression has been reported in ageing human skin fibroblasts and ECs but also after exposure to ROS, however, more specific roles in the induction of senescence have not been elucidated [103].

MiR-200 family, overexpressed in ageing EC, could participate in senescence pathophysiology by modulating apoptosis and endothelial-mesenchymal transition as well as ROS production.

### 4.7. miR-146a

MiR-146a can be detected and quantified in various human body fluids and its concentrations differ in cancer patients, potentially reflecting cancer subtype or disease stage, rendering it a very attractive non-invasive biomarker for diagnosis establishment and controlling for disease progression [104]. The role of miR-146a in the regulation of immune response, predominantly of the innate immune system, has been extensively investigated. Available evidence provides a strong argument for investigating its role in the pathophysiology of autoimmune and neurodegenerative diseases [105]. The innate immune response is initiated by the activation of Toll-like receptors (TLRs), leading to the activation of several signaling pathways with downstream activation of NF-κB, mitogen-activated protein kinases (MAPKs), members of the interferon regulatory factor family and activator protein1 (AP-1) [106].

Interestingly, the expression of miR-146a is under the complex mechanism of self-limiting control. Lipopolysaccharides, TLR4 agonists, induce miR-146a expression and the modulation of downstream signaling pathways in a negative feedback manner, thus reducing the expression of the miRNA itself and limiting the production of other inflammatory molecules in order to tightly regulate the extent of inflammatory response and prevent overstimulation [105].

MiR-146 is also reported to regulate vascular inflammation and to control the intensity and duration of endothelial activation in response to cytokine stimulation. Levels of miR-146a increase in the later stages of the inflammatory response (when most other genes involved in the inflammatory response are downregulated) and remain elevated for several days, even in the absence of pro-inflammatory cytokines [63]. The expression of miR-146a in endothelial and smooth muscle cells is also reported to increase in response to shear stress, thus preventing neointimal proliferation. This effect is probably mediated by β1 and β3 integrins and by Nrf2 [63]. MiR-146a expression was reported to be increased in senescent cells, supposedly to counteract the increased inflammation associated with ageing [107,108]. To date, there is no evidence of miR-146a involvement in the mechanisms of cellular response to oxidative stress in both young and senescent cells [107,108].

MiR-146a, overexpressed in senescent EC, could play an important role in the development of endothelial dysfunction by modulating vascular inflammation and neointimal proliferation.

### 4.8. miR-217

In vitro studies have recognised miR-217 as one of the most expressed miRNAs in ageing human ECs. The level of circulating miR-217 could serve as a biomarker of ageing and is reported to be associated with increased CV risk [65]. The expression of miR-217 was found to be increased in senescent cells, in parallel to SIRT1 downregulation [109]. MiR-217 is reported to regulate cell cycle, differentiation, proliferation, and senescence induction by the downregulation of SIRT1 and downstream signaling pathways including FoxO3 and p53 [64]. Interestingly, metformin, known to modulate SIRT1, was reported to change the expression of numerous miRNAs, including miR-217 although in a “U-shaped manner” [110].

To date, overexpression of miR-217 has been found in different types of senescent cells, whereas the downregulation of its expression is reported to partially reverse the senescent phenotype [110]. One of the main mechanisms responsible for the induction of senescence is the suppression of DNMT1-mediated methylation of p16 and pRb [111]. Other reported mechanisms illustrate a wide spectrum of functions controlled by one single miRNA: downregulation of signaling pathways leading to eNOS activation (e.g., VEGF), reduced NO production and signaling pathways responsible for maintaining the structure and function of extracellular matrix, collagen and actin [65]. Overexpression of endothelial miR-217 in apoE^−/−^ mice was reported in association with some of the most common CV disorders: endothelial dysfunction, increased blood pressure, development of atherosclerosis, coronary artery disease, and left ventricular systolic and diastolic dysfunction [65]. The same study revealed an excellent association between plasma levels of circulating miR-217 with conventional CV risk factors (low physical activity, poor dietary habits, high BMI). In healthy individuals, plasma levels of miR-217 were highly associated with CV risk factor score, emerging as a novel biomarker for CV risk assessment [65].

To conclude: miR-217, overexpressed in senescent cells, has been reported to regulate some of the most important aspects in senescence pathophysiology: cell cycle, differentiation, proliferation and senescence induction as well as NO production and maintenance of ECM structure and function.

### 4.9. miR-181b

The human and mouse miR-181 family contains four members (miR-181a, miR-181b, miR-181c, and miR-181d) encoded by three different transcripts located on different chromosomes [112]. Regardless of them belonging to the same family, these miRNAs could have similar or distinct seed sequences and target genes. Since -5p and -3p miR-181s have different seed sequences, they probably target different genes and signaling pathways. On the other hand, despite all the -5p miR-181 family members sharing the same seed sequence, they target different genes [112]. The main role of miR-181b is based on its involvement in regulating the inflammatory response. The expression of miR-181b in ECs was reported to be reduced in response to pro-inflammatory stimuli such as TNF-α or LPS. In turn, overexpression of miR-181b was reported to suppress genes for adhesion molecules VCAM-1 and E-selectin, and reduced leukocyte adhesion to activated endothelium [66]. MiR-181b is a potent regulator of downstream (but not upstream) NF-κB signaling, probably by targeting importin-α3, a protein critical for NF-κB translocation from the cytoplasm to the nucleus [66]. To date, no major effect of miR-181b on other MAPK downstream signaling pathways (ERK1/2, p38, or JNK) has been reported.

The expression of miR-181b in cardiac fibroblast was markedly reduced after exposure to angiotensin II, suggesting its potential role in hypertension and cardiac fibrosis [113]. Overexpression of miR-181b has a protective role against endothelial-mesenchymal transition, the development of pulmonary arterial hypertrophy, pulmonary hypertension and right ventricular remodelling in rats [67]. This effect is probably mediated by targeting the TGFRB1 gene [67]. Finally, it was found that miR-181b reduces the inflammation-induced expression of tissue factor and generation of activated factor X, thus having a protective role against the increased thrombogenicity observed in patients with diabetes [114]. The reduction of its expression was also observed in obese patients, so it is now considered a novel potential biomarker of metabolic syndrome and coronary artery disease [115]. Increased expression of miR-181b was observed in peripheral blood mononuclear cells in patients with DM type 2 taking resveratrol enriched grape extract for 12 months, in parallel with the reduced expression of pro-inflammatory cytokines (CCL3, IL-1β and TNF-α) [116].

Evidence of its role in endothelial senescence is limited. Available data show increased expression of miR-181b in ageing HUVEC. Overexpression was also reported in aortic valve ECs after low-magnitude bidirectional shear stress and after exposure to oxidative stress. Overexpression of miR-181b could promote extracellular matrix degradation, supposedly by inhibiting the tissue inhibitor of metalloproteinase 3 (TIMP3) [68].

Thus, miR-181b, overexpressed in senescent cells, could participate in the development of endothelial dysfunction by modulation of leukocyte adhesion, inflammatory response, endothelial-mesenchymal transition and extracellular matrix degradation.

### 4.10. let-7 Family

The let-7 family of miRNAs is considered to be one of the largest and most conserved families of miRNAs across different species. The family comprises 10 mature let-7 miRNAs: let-7a, let-7b, let-7c, let-7d, let-7e, let-7f, let-7g, let-7i, miR-98 and miR-202, encoded by 13 precursor genes located on different chromosomes [117]. The let-7 family is regulated by the LIN28 RNA-binding protein, which regulates the production of let-7 at the post-transcriptional level, which in turn suppresses LIN28 at the post-transcriptional level [69].

Available evidence supports a complex role for LIN28 in cellular growth and metabolism, given that it has been reported to: upregulate many cell cycle regulators (MYC, Ras, HMG2A) through let-7, including upregulation of the IGF-PI3K-mTOR signaling axis, stimulation of the translation of mRNAs encoding cyclin A/B, and cyclin-dependent kinases (Cdk2, Cdk4, Cdc2, and Cdc20), and promotion of cellular growth by promoting the ribosomal synthesis of proteins [69,70,71]. In cancer cells, the let-7 family has a tumour suppressing role as it inhibits tumour growth and metastasis development [117]. Given that the let-7 family is downregulated or lost in many human cancers, the restoration of its expression emerges as an attractive therapeutic option for cancer treatment [118]. The let-7 family has been found to be highly expressed in different CV cell types: endothelial, smooth muscle cells, and cardiomyocytes. Overexpression of different members of the let-7 family is a common feature in myocardial ischemia, infarction, ischemic cardiomyopathy, cardiac fibrosis, and congestive heart failure [119]. A dramatic decrease in plasma level of let-7b is observed 4–12 h before onset of acute myocardial infarction, reaching its peak level at 8 h, similar to the peak of cardiac troponin I [120]. Let-7g is considered to have an atheroprotective role, as it inhibits the uptake of oxidised-low density lipoprotein (ox-LDL) into endothelial and vascular smooth muscle cells [121]. Evidence of its involvement in different pathophysiologic processes (inflammation, monocyte adhesion, thrombosis, proliferation, migration, angiogenesis, autophagy and apoptosis) suggests its complex and important role in CV homeostasis. The effects are mediated by several overlapping regulatory pathways, but the most important effects are achieved through TGF-b and SIRT-1 signaling pathways [72].

Let-7g was reported to be involved in the endothelial–mesenchymal transition, an important pathophysiologic mechanism in cardiac fibrosis [119]. The exact mechanism for this effect is not known. After similar expression alterations were found in both failing human hearts and foetal hearts, it was proposed that miRNAs could contribute to these pathologies by reactivation of a foetal gene programme. In perspective, the development of new drugs to specifically target miRNAs involved in these processes would be a breakthrough in the treatment of various CV diseases [122].

Thus, the let-7 family, reported to be upregulated in senescent cells, could play one of the key roles in senescence pathophysiology by regulating some of the key signalling pathways in cellular growth and metabolism, as well as the cell cycle.

### 4.11. miR-17-92 Cluster

The miR-17-92 cluster comprises seven miRNAs: miR-17-3p, miR-17-5p, miR-18a, miR-19a, miR-19b, miR-20a, and miR-92a. After the ectopic expression of a truncated version of the cluster (lacking miR-92) had been found in B-cell lymphoma, this cluster was supposed to have an oncogenic character and was named the first ‘oncomiR’ (oncomiR-1) [123].

Expression of the miR-17/92 cluster is often deranged in hematopoietic malignancies and solid tumours [123]. Overexpression of miR-17-92 promotes cell cycle progression and proliferation, inhibits apoptosis, and induces tumour angiogenesis [74]. The mechanism behind this is, at least partially, the inhibition of oncogenic, Ras-induced senescence, which is mediated by the p38 MAPK [73]. Research performed on an animal model of ageing-associated heart failure confirmed that miR-17-92 could disrupt oncogenic Ras-induced senescence in primary human fibroblasts, by directly targeting p21 [74]. However, evidence of its role in the induction of non-oncogenic senescence is still limited. MiR-17, miR-19b, miR-20a, and miR-106a were found to be downregulated in senescent HUVECs, probably in response to an increased level of cdk inhibitor p21/CDKN1A [124].

Members of this cluster have various positive effects on the CV system beyond those observed in tumours. MiR-17-92 was reported to be essential for cardiac development, both embryonic and postnatal [75]. This effect is mediated by VEGF through mitogen-activated protein kinase (MAPK) activation and the Elk-1 phosphorylation signaling pathway [76]. MiR17, miR-19, and miR-92 may directly inhibit apoptosis through MAPK/ERK and PI3 K/Akt signaling pathways [77]. MiR-17 expression was reported to be higher in patients with coronary disease compared to healthy individuals, and was in positive correlation with levels of triglycerides, LDL-cholesterol and ApoB. Conversely, expression of miR-92a was lower in patients with coronary disease and positively correlated with HDL-cholesterol and ApoA-1 [125]. This makes the miR-17-92 cluster an interesting potential biomarker for coronary disease.

MiR-17-92 cluster, downregulated in senescent cells, could play an important role in senescence pathophysiology by modulating some of the key steps in cellular biology: cell cycle progression and proliferation and apoptosis.

## 5. Perspectives and Future Directions

Knowledge regarding the role of miRNAs in endothelial function and EC senescence has increased substantially within the last few years. However, the complex regulatory pattern of miRNAs is still insufficiently understood. Undoubtedly, miRNAs play a significant role in ECs, including the process of senescence, since miRNAs essentially participate in complex control of the fine balance between stimulatory and inhibitory signals with consequent numerous molecular and cellular events that occur dynamically in ECs and are impaired by ageing. Thus, dysregulation of miRNAs play an important role in EC senescence. While the expression of some miRNA is increased, the expression of others is decreased. Furthermore, miRNAs can be overexpressed as the pathological response or as the beneficial compensatory mechanisms; the same applies to the downregulation of miRNAs.

Overall, miRNAs could serve as both markers of endothelial senescence and as targets for possible intervention aiming to reduce senescence and retard the ageing of the endothelium. Whereas the clinical applications of miRNAs as circulating markers of EC senescence seems plausible in the near future, miRNA-based therapies of ECs via cells reprogramming that targets several pathways (and not only one gene as with small interfering RNA (siRNA) therapy) represent a very attractive approach but are still subject of extensive research. One of the biggest challenges of such an approach is the fact that one single miRNA could target multiple genes thus having multiple potential side-effects on remoted tissues and organs.

Currently, the most promising results are expected from studies performed in the treatment of different types of tumours. The evidence of effective miRNA-based therapy for endothelial dysfunction is lacking, but emerging evidence shows that this therapy could have a positive impact on some features of endothelial dysfunction as well as some conventional CV risk factors. In vivo study using a mouse model of pressure-overload-induced heart disease showed that using miR-21 antisense oligonucleotides to lower miR-21 level in activated fibroblasts reduces the extent of heart fibrosis and improves heart function [126]. A similar study, performed on a mouse model of abdominal aortic aneurysm, showed that lowering of miR-21 level is associated with an increase in aneurysm diameter [127]. Thus, an effective approach to prevent aneurysm progression should result in miR-21 overexpression, keeping in mind that this could also have serious side effects, such as the increased risk of hepatocellular carcinoma development or increased myocardial fibrosis [127].

In order to better understand the pathophysiology of endothelial dysfunction, significant overlap between senescence and expression to CV risk factors as well as the overlap between CV diseases and age-related diseases should be taken into account. Thus, the concomitant analysis including functional endothelial methods (flow-mediated dilatation of brachial artery), circulating markers of endothelial (dys)function (adhesion and inflammatory molecules and von Willebrand factor) along with the assessment of a wide range of miRNAs in young, middle-aged and old subjects, in three distinct groups (subjects with established CV diseases, subjects with CV risk factors, and in healthy subjects) may be a promising approach that could serve as valuable wide network data that could be helpful for the identification of the clinically most relevant miRNAs and their interactions in the process of senescence. Such studies are already underway. Furthermore, overlapping as well as causal and reversal causal relationships between senescence and CV risk factors and diseases could thereby also be further explored. Importantly, it can be expected that knowledge, translated from intensive research of miRNA in tumour development and progression and their role as both markers and therapeutic targets, would be beneficial for understanding the role of miRNAs in EC senescence and ageing. Taking all these facts together, it seems reasonably optimistic to expect that miRNA research will make important progress in our understanding of EC senescence and ageing, as well as the development and progression of associated age-related diseases.

## Figures and Tables

**Table 1:** Potential mechanism by which particular miRNA could take part in senescence pathophysiology and its target genes/signalling pathways by which the effect is achieved. HUVEC—primary human umbilical vein endothelial cells; HAVEC—primary human aortic valve endothelial cells; HAEC—primary human aortic endothelial cells; HPASMC—human pulmonary artery smooth muscle cells; HPAEC—human pulmonary artery endothelial cells; ECM—extracellular matrix.

miRNA	Main Effect	Target Genes	Target Signalling Pathways	Type of Study	Reference
miR-126	inhibition of angiogenesis and tumour growth		PI3K/Akt	4T1 cell line (spontaneous metastatic mammary carcinoma)	[38]
	anti-inflammatory, antioxidative		SIRT1/Nrf2	HUVEC	[39,40]
	reduced expression of VCAM-1 in ECs, reduced leukocyte adhesion to EC	VCAM-1		HUVEC	[41,42]
	modulation of migration and formation of vessel lumen	VEGF-A		HUVEC, 4T1 cell line (spontaneous metastatic mammary carcinoma)	[38,42]
	Regulation of gene expression in: metabolic control, oxidized phospholipid signaling cellular response to hypoxic, oxidative and electrophilic stress repair and degradation of damaged macromolecules	Nrf2		HAEC	[43]
miR-21 and miR-100	angiogenesis, proliferation and increased metabolic activity of EC		VEGFA/MYC	HUVEC	[29]
miR-21	mitochondrial functioning		AMPK-p53/p16	HUVEC	[44]
	reduction of endothelial/mesenchymal transition	kallistatin/TGF-β1		endothelial progenitor cells, HUVEC	[45,46]
	reduction of oxidative stress	eNOS, SIRT1 and FoxO1		endothelial progenitor cells, HUVEC	[45,46]
miR-100	inhibition of proliferation of EC and migration of VSMC		mTOR	HUVEC	[47]
	attenuated atherogenesis and decreased plaque area			HUVEC	[48]
miR-34a	deacetylation of regulatory proteins and transcription factors involved in senescence signalling pathways	SIRT1, CCNA2, eNOS	p53, NF-κB, PPARγ, FoxO1	HUVEC, rat aortic EC	[49,50,51]
	increased NO synthesis	eNOS/SIRT	Akt	HUVEC, rat aortic EC	[51]
	inhibition of PCSK9 expression/regulation of thrombosis and fibrinolysis, glucose metabolism and inflammation	SIRT1, FoxO1		neonatal rat cardiomyocytes, mouse cardiomyocytes,	[52]
miR-130a	increased proliferation and migration of EC	HOXA5, MEOX2		Mouse aortic EC	[53]
	angiogenesis, NO bioavailability	MEOX2/eNOS/VEGF	Akt	HUVEC	[54]
	enhanced hypoxia-induced smooth muscle cell proliferation	BMPR2	p21	HPASMC, HPAEC	[55]
	modulated expression of fibrotic and inflammatory genes and adhesion molecules	PPARγ/collagens, Fn and CTGF		neonatal rat cardiac fibroblasts	[56]
miR-222-221 cluster	anti-proliferative, anti-migratory and pro-apoptotic effects in EC (opposite in VSMC)	P27, p57		rat aortic EC and VSMC culture	[57]
	increased cardiomyocyte proliferation	HIPK1, homeobox-1		primary neonatal rat cardiomyocytes	[58]
	cell cycle regulation/cellular growth and division; autophagy/apoptosis	p27, p57, PTEN, TIMP3	mTOR	primary neonatal rat cardiomyocytes	[59]
	supressed NO synthesis, increased production of ROS	PPARγ/coactivator-1α	NF-κB	HAEC, HUVEC	[60]
miR-200 family	regulation of endothelial-mesenchymal transition	ZEB1, ZEB2		HAEC	[61]
	increased ROS production, induction of apoptosis	SIRT1, p53		HUVEC	[62]
miR-146a	regulation of vascular inflammation	IL-1R, IL-6R, Nrf2	NF-κB	human aortic ECs and SMCs cultures	[63]
	inhibition of neointimal proliferation	β1 and β3 integrins, Nrf2		human aortic ECs and SMCs cultures	[63]
miR-217	regulation of cell cycle, differentiation, proliferation and senescence induction	SIRT1	FoxO3, p53		[64]
	eNOS inhibition		APLNR, VEGF, adenylyl cyclase, LPAR	primary Mouse Lung Endothelial Cell	[65]
	maintenance of structure and function of ECM, collagen and actin			primary Mouse Lung Endothelial Cell	[65]
miR-181b	regulation of inflammatory response		NF-κB	HUVEC	[66]
	reduced leukocyte adhesion to EC	VCAM-1, E-selectin		HUVEC	[66]
	inhibition of endothelial-mesenchymal transition		TGFRB1	rat pulmonary arterial ECs line	[67]
	promotion of ECM degradation		TIMP3	HAVEC	[68]
let-7 family	regulation of cell cycle/cellular growth	LIN28/MYC, Ras, HMG2A, cyclin A/B, cyclin-dependent kinases (Cdk2, Cdk4, Cdc2, and Cdc20)	IGF-PI3K-mTOR	primary cortical neurons mouse myoblasts cell culture	[69,70,71]
	reduced uptake of ox-LDL into ECs and VSMCs		TGF-b SIRT-1	HUVEC	[72]
miR-17-92 cluster	promotes cell cycle progression and proliferation inhibits apoptosis induces tumour angiogenesis	Ras oncogene	p38 MAPK	primary human fibroblasts	[73]
	disruption of Ras induced senescence	p21		primary human fibroblasts	[74]
	cardiac development (embryonic and postnatal)	VEGF/MAPK	Elk-1	mouse embryonic stem cells culture	[75,76]
	inhibition of apoptosis		MAPK/ERK PI3 K/Akt	mantle cell lymphoma cell line	[77]
